# Relationships Between Spinal Alignment and Muscle Mass in Osteoporosis Patients Over 75 Years of Age Who Were Independent and Maintained Their Activities of Daily Living

**DOI:** 10.7759/cureus.15130

**Published:** 2021-05-19

**Authors:** Ayumu Kawakubo, Masayuki Miyagi, Hisako Fujimaki, Gen Inoue, Toshiyuki Nakazawa, Takayuki Imura, Wataru Saito, Kentaro Uchida, Seiji Ohtori, Masashi Takaso

**Affiliations:** 1 Orthopaedic Surgery, Kitasato University School of Medicine, Sagamihara, JPN; 2 Orthopaedic Surgery, Kitasato University, Sagamihara, JPN; 3 Orthopaedics, Chiba University Hospital, Chiba, JPN; 4 Orthopaedic Surgery, Graduate School of Medicine, Chiba University, Chiba, JPN

**Keywords:** spinal sagittal alignment, bone mineral density, muscle muss, osteoporosis, low back pain

## Abstract

Introduction

Elderly patients with osteoporosis often complain of back pain associated with pathological vertebral fractures caused by abnormal spinal alignment. Few reports evaluate the relationships among muscle mass, bone mineral density (BMD), sagittal spinal alignment, and low back pain. We hypothesized that decreasing muscle mass in elderly patients with osteoporosis could cause spinal alignment abnormalities. The aim of the current study were to compare the characteristics between spinal sagittal normal alignment and malalignment and to evaluate the relationships between sagittal spinal alignment and muscle mass in elderly patients with osteoporosis.

Methods

Fifty patients aged 75 years or more (mean age = 80.5 years) with osteoporosis were included in this study. We evaluated the sagittal vertical axis (SVA), pelvic tilt (PT), pelvic incidence minus lumbar lordosis (PI-LL), the number of vertebral fractures (N of VFs), BMD by dual-energy X-ray absorptiometry, and trunk and skeletal muscle mass using bioelectrical impedance. Low back pain was evaluated using the Oswestry Disability Index (ODI). Corrected trunk muscle mass (trunk muscle mass index, TMI) and corrected limb muscle mass (skeletal mass index, SMI) also were measured. Patients were divided into two groups for comparison: a ‘normal’ group and a sagittal spinal ‘malalignment’ group. Multiple regression analysis was carried out to evaluate the relationship between spinal sagittal parameters and muscle mass.

Results

Comparisons between normal and malalignment groups for SVA, N of VFs, BMI, and SMI showed significantly higher in the malalignment group versus the normal group (p < 0.05). N of VFs, BMI, and TMI, for PT, and BMI, TMI, SMI, and ODI scores for PI-LL showed significantly higher in the malalignment group versus the normal group (p < 0.05). There were significantly more vertebral fractures in the malalignment group than in the normal group (p < 0.05). However, there were no significant differences of pure muscle mass between the two groups. When adjusted by BMD and the number of vertebral fractures, SMI and TMI were positively correlated to PI-LL and SVA (p < 0.05).

Conclusion

Elderly patients with osteoporosis and a sagittal spinal malalignment had more vertebral fractures and a higher risk of low back pain than patients with normal spinal alignment. Patients with a sagittal spinal malalignment who were independent and maintained their activities of daily living (ADL) showed high BMI and maintained muscle mass, independent of BMD and the N of VFs, contrary to our hypothesis.

## Introduction

Elderly patients with osteoporosis often complain of back pain associated with pathological vertebral fractures which cause abnormal spinal alignment. Abnormal spinal alignment causes a decrease in the quality of life and activities of daily living (ADL). Several spinal surgeons have increasingly focused on the relationship between abnormal spinal alignment and low back pain and corrective surgeries for spinal malalignments have been increasing in recent years. Further, several authors have reported a sagittal spinal malalignment as one of the risk factors for low back pain (LBP) [1,2].

Curtis et al. report that the loss of muscle and bone due to aging threatens the loss of independence in later life [3]. Several authors have reported that bone mineral density (BMD) is highly correlated with muscle mass in osteoporosis patients [4,5]. On the other hand, other authors have reported that spinal deformity patients usually have low muscle volume and poor muscle quality [6,7]. However, few reports evaluate the relationships among muscle mass, BMD, sagittal spinal alignment, and low back pain in osteoporosis patients who did not require special treatment for spinal deformity, including surgical treatment. We hypothesized that lower muscle mass in elderly osteoporosis patients who did not require treatment for spinal deformity also could cause abnormalities in spinal alignment. The aim of the current study was to compare the characteristics between spinal sagittal normal alignment and malalignment and to evaluate the relationships between sagittal spinal alignment and muscle mass in elderly patients with osteoporosis.

## Materials and methods

Subjects

A total of 50 patients aged 75 years or more with osteoporosis (all women, mean age: 80.2 years; 75-90 years) were included in this study. All patients could come to the outpatients department by themselves. Patients who need any help in daily livings were excluded.

Measurements

In all cases, we evaluated the sagittal vertical axis (SVA), pelvic tilt (PT), and pelvic incidence minus lumbar lordosis (PI-LL) using a lateral whole-spine radiograph of patients in a standing position for the measurement of sagittal spinal alignment. BMD of the lumbar spine (LS) and femoral neck (FN) was measured using dual-energy X-ray absorptiometry analysis, and trunk and skeletal muscle mass was evaluated using bioelectrical impedance analysis (TANITA MC-780A). LBP was evaluated using the Oswestry Disability Index (ODI). Trunk and skeletal muscle mass corrections were measured by dividing body weight by body height squared. Corrected trunk muscle mass (trunk muscle mass index, TMI) and corrected skeletal muscle mass (skeletal muscle mass index, SMI) were also measured.

Statistical analysis

Based on the Scoliosis Research Society-Schwab adult spinal deformity classification, we divided the patients into two groups for comparison, a ‘normal’ group and a sagittal spinal ‘malalignment’ group that included patients with a PI-LL of 10 degrees or more, an SVA of 4 cm or more, or a PT of 20 degrees or more. The Leven's test was used to assess the equality of variance of variables of interest. For variables with unequal variances, the Mann-Whitney U-test was applied. For variables with equal variances, an unpaired t-test was used. Multiple regression analysis was carried out to evaluate relationships between spinal sagittal parameters and muscle mass. The objective variables were SVA, PT, and PI-LL, and their association with TMI or SMI was analyzed using nonlinear regression analysis after the data were adjusted for the number of vertebral fractures and BMD. When we evaluated the relationships between spinal parameters and TMI, the data were adjusted by LS BMD; for SMI, the data were adjusted by FN BMD. Statistical tests were considered significant at p < 0.05, and all tests were two-tailed.

## Results

Patients were of mean age 80.3 years and had an average of 3.4 vertebral bodies fractured. The details of characteristics of patients in the current study were shown in Table 1.

**Table 1:** The characteristics of patients. SVA: sagittal vertebral axis; PT: pelvic tilt; PI-LL: pelvic incidence minus lumbar lordosis; LS: lumbar spine; FN: femoral neck; BMD: bone mineral density; N of VFs; the number of vertebral fractures; ODI: Oswestry Disability Index; BMI: body mass index; TM: trunk muscle mass; SM: skeletal muscle mass; TMI: corrected trunk muscle mass; SMI: corrected skeletal muscle mass.

	Mean	SD
Age(years)	80.2	3.6
SVA	63.6	55.9
PT	27.5	11.4
PI-LL	14.7	19.9
LSBMD (g/cm^2^)	0.685	0.166
FNBMD (g/cm^2^)	0.492	0.103
N of VFs	3.3	3.4
ODI (%)	32.4	21.1
Body height (cm)	145.3	6.4
Body weight (kg)	44.1	7.8
BMI (kg/m^2^)	20.6	3.8
TM (kg)	17.6	1.9
SM (kg)	12.2	1.9
TMI (kg/m^2^)	8.3	0.6
SMI (kg/m^2^)	5.7	0.8

Comparisons between the normal group and the malalignment group for SVA, PT and PI-LL are summarized in Table 2.

**Table 2:** comparisons of the sagittal vertebral axis (SVA) or pelvic tilt (PT) or the pelvic incidence minus lumbar lordosis (PI-LL) between the ‘normal’ group and the sagittal spinal ‘malalignment’ group LS: lumbar spine; FN: femoral neck; BMD: bone mineral density; N of VFs: the number of vertebral fractures; ODI: Oswestry Disability Index; BMI: body mass index; TM: trunk muscle mass; SM: skeletal muscle mass; TMI: corrected trunk muscle mass; SMI: corrected skeletal muscle mass.

		Normal group		Malalignment group		p-value
		Mean	SD	Mean	SD	
SVA	N	19		31		-
	Age (years)	79.1	2.6	80.9	4.0	0.066
	LSBMD (g/cm^2^)	0.665	0.164	0.698	0.169	0.500
	FNBMD (g/cm^2^)	0.500	0.088	0.488	0.112	0.687
	N of VFs	1.9	2.5	4.1	3.6	0.026
	ODI (%)	24.6	19.0	37.1	21.3	0.054
	Body height (cm)	147.6	4.7	144.1	6.8	0.077
	Body weight (kg)	42.3	7.7	45.0	7.8	0.253
	BMI (kg/m^2^)	18.8	3.2	21.7	3.8	0.008
	TM (kg)	17.8	1.7	17.5	2.0	0.598
	SM (kg)	12.0	1.7	12.2	1.9	0.621
	TMI (kg/m^2^)	8.1	0.7	8.5	0.6	0.063
	SMI (kg/m^2^)	5.4	0.6	5.9	0.8	0.018
PT	N	11		39		-
	Age (years)	79.5	2.7	80.4	3.9	0.447
	LSBMD (g/cm^2^)	0.678	0.172	0.688	0.167	0.865
	FNBMD (g/cm^2^)	0.462	0.091	0.501	0.106	0.279
	N of VFs	1.0	1.8	3.9	3.5	0.001
	ODI (%)	23.7	17.6	34.9	21.6	0.141
	Body height (cm)	147.5	4.3	144.8	6.7	0.295
	Body weight (kg)	41.4	6.4	44.6	8.0	0.299
	BMI (kg/m^2^)	18.2	3.3	21.3	3.7	0.017
	TM (kg)	17.6	1.7	17.6	2.0	0.923
	SM (kg)	12.4	1.6	12.1	1.9	0.743
	TMI (kg/m^2^)	8.0	0.8	8.4	0.6	0.045
	SMI (kg/m^2^)	5.5	0.7	5.8	0.8	0.235
PI-LL	N	24		26		-
	Age (years)	79.8	2.5	80.6	4.5	0.397
	LSBMD (g/cm^2^)	0.699	0.148	0.673	0.184	0.596
	FNBMD (g/cm^2^)	0.493	0.093	0.492	0.113	0.987
	N of VFs	2.3	3.0	4.2	3.5	0.052
	ODI (%)	26.0	18.4	39.1	22.2	0.038
	Body height (cm)	147.5	4.5	143.5	7.2	0.033
	Body weight (kg)	43.5	7.9	44.6	7.9	0.635
	BMI (kg/m^2^)	19.4	3.5	21.7	3.9	0.035
	TM (kg)	8.1	0.6	8.5	0.6	0.683
	SM (kg)	5.5	0.6	5.9	0.8	0.929
	TMI (kg/m^2^)	17.8	1.7	17.5	2.1	0.014
	SMI (kg/m^2^)	12.1	1.7	12.2	2.0	0.034

Comparing the normal and malalignment groups based on SVA, the BMI and SMI were significantly higher and more vertebral fractures were present in the malalignment group compared with the normal group (p < 0.05). In contrast, there were no significant differences in the age, BMD, body height, body weight, TM, SM, TMI and ODI scores between the normal and malalignment groups. In terms of PT, the BMI and TMI were significantly higher and there were more vertebral fractures in the malalignment group compared with those in the normal group (p<0.05). Significant differences were not evident for the age, BMD, body height, body weight, TM, SM, SMI, or ODI scores between the two groups. With respect to PI-LL, the BMI, TMI, SMI, and the ODI scores were significantly higher in the malalignment group compared to the normal group and body height was significantly lower in the malalignment group versus the normal group (p < 0.05). In contrast, there were no significant differences age, BMD, body weight, TM, and SM between the two groups.

TMI was positively correlated with the SVA (p < 0.05) and PI-LL (p < 0.05) when adjusted by the number of vertebral fractures and the LS-BMD. SMI also was correlated positively with the SVA (p < 0.05) and PI-LL (p < 0.05) when adjusted by the number of vertebral fractures and the FN-BMD. In addition, BMI was positively correlated with the SVA (p < 0.05), PT (p < 0.05) and PI-LL (p < 0.05) when adjusted by the number of vertebral fractures and the LS-BMD (Table 3).

**Table 3:** Multiple regression analysis to evaluate relationships between spinal sagittal parameters and muscle mass. SVA: the sagittal vertebral axis; PT: pelvic tilt; PI-LL: the pelvic incidence minus lumbar lordosis; N of VFs: the number of vertebral fractures; BMD: bone mineral density; LS: lumbar spine; FN: femoral neck; TMI: corrected trunk muscle mass; SMI: corrected skeletal muscle mass; BMI: body mass index.

		Parameter estimate	Standard error	t-value	p-value
SVA	N of VFs	2.594	2.361	1.100	0.277
	LS BMD	68.756	47.298	1.454	0.153
	TMI	26.867	12.379	2.170	0.035
PT	N of VFs	0.821	0.493	1.666	0.103
	LS BMD	-4.376	9.879	-0.443	0.660
	TMI	3.994	2.585	1.545	0.129
PI-LL	N of VFs	0.868	0.855	1.016	0.315
	LS BMD	9.588	17.121	0.560	0.578
	TMI	9.941	4.481	2.219	0.032
SVA	N of VFs	2.735	2.375	1.152	0.256
	FN BMD	11.342	77.717	0.146	0.885
	SMI	24.120	10.349	2.331	0.024
PT	N of VFs	1.055	0.478	2.208	0.032
	FN BMD	11.460	15.640	0.733	0.468
	SMI	3.631	2.083	1.743	0.088
PI-LL	N of VFs	1.046	0.846	1.236	0.223
	FN BMD	-1.802	27.686	-0.065	0.948
	SMI	8.351	3.687	2.265	0.028
SVA	N of VFs	0.274	0.129	4.464	0.035
	LS BMD	1.171	2.168	0.292	0.589
	BMI	0.295	0.126	5.534	0.019
PT	N of VFs	0.530	0.242	4.810	0.028
	LS BMD	0.185	2.549	0.005	0.942
	BMI	0.318	0.144	4.905	0.027
PI-LL	N of VFs	0.160	0.103	2.436	0.119
	LS BMD	-1.628	2.120	0.590	0.443
	BMI	0.228	0.112	4.173	0.041

## Discussion

The patients in this study had osteoporosis as demonstrated by the average number of vertebral bodies fractured. In this study, contrary to our hypothesis, muscle mass corrected by body height and BMI were tended to be high in patients with spinal sagittal malalignment, but pure muscle mass were not significantly different between the two groups. Alternatively, there were higher LBP scores and more vertebral fractures among patients with a sagittal spinal malalignment than among those patients classified as normal.

In the current study, there were higher LBP scores reported by patients with a sagittal spinal malalignment. Takemitsu and coworkers stated that 95% of patients with lumbar kyphosis reported low back pain as well as severe disruption to their ADL and raised these issues regarding kyphosis [8]. A meta-analysis reported by Chun and colleagues showed that LBP was strongly correlated with a decreasing lumbar lordosis indicating that a sagittal spinal malalignment was likely implicated in comparison with age-matched healthy controls [9]. These findings suggest that LBP was strongly correlated with sagittal spinal alignment.

Rafael Menezes-Reis et al. have reported that spinopelvic parameters show a correlation with lumbar muscle volumes [10] among patients with sagittal spinal alignment who report LBP. In addition, Enomoto et al. report that patients with a sagittal spinal malalignment have severe muscle fatigue in the upper lumbar spine [11]. Therefore, LBP due to a sagittal spinal malalignment appears to be correlated with muscle mass.

**Figure 1:**
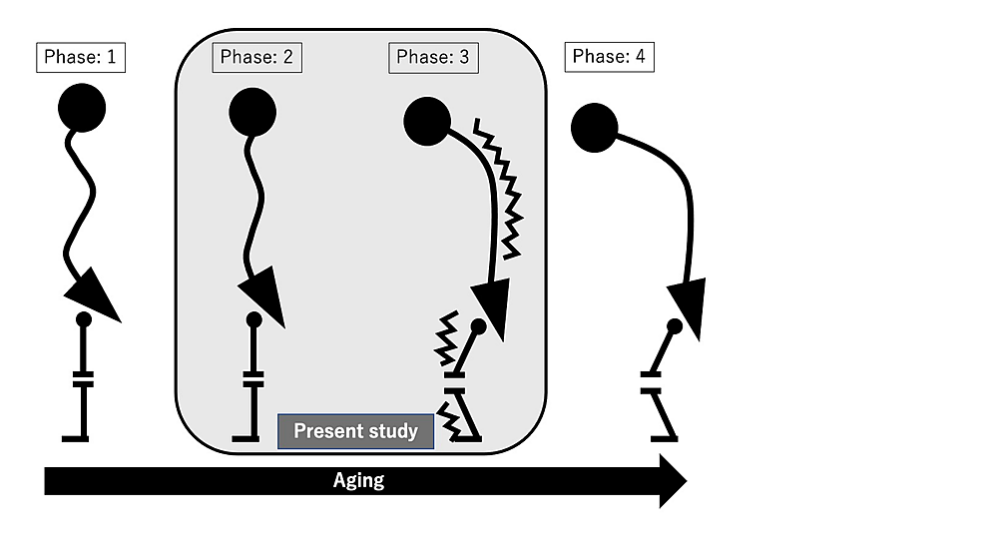
The hypothesis of four phases of aging and spinal alignment: normal phase (phase 1), early phase (phase 2), compensation phase (phase 3), and terminal phase (phase 4).

Interestingly, in the current study patients with a sagittal spinal malalignment tended to have a higher muscle mass, contrary to our hypothesis. However, Roseline D`hooge et al. have reported that lumbar muscle degeneration is a feature of LBP and is characterized by a decrease in muscle size [12]. Furthermore, Yagi and colleagues have reported that the cross-sectional area of the multifidus and psoas muscles were significantly lower in patients with degenerative lumbar scoliosis than in patients with lumbar spinal stenosis [13]. Eguchi et al. have also reported that truncal muscle mass was significantly lower in patients with degenerative lumbar scoliosis who were scheduled for corrective surgery than in patients with lumbar spinal stenosis [14]. The previous reports suggest that a loss of muscle mass might be implicated in the progression of spinal deformities and back pain. However, a discrepancy exists between the patients in the previous reports we cite and those in the present study. In this study, muscle mass corrected by body height were tended to be higher in malalignment group compared with normal group, but pure muscle mass were no significant differences between two groups. These findings indicated correction methods of muscle mass might lead to these discrepancies. Regarding the correction methods of muscle mass, several authors evaluated using muscle mass corrected by body height and body weight as well as pure muscle mass [15-17]. In the correction by body height, patients with VFs might be underestimated body height, and overestimated muscle mass. Therefore, which correction methods are appropriate would be still controversial. However, these findings indicated muscle mass were at least maintain in spinal sagittal malalignment group. Further, elderly patients with osteoporosis who were independent and maintained their ADL despite LBP were included in the present study compared with those in previous study. Enomoto et al. have reported that patients with a sagittal spinal malalignment had greater muscle activity in the lower back in a standing position [11]. Based on these findings, we hypothesized that there were four phases of aging and spinal alignment: normal phase (phase 1), early phase (phase 2), compensation phase (phase 3), and terminal phase (phase 4) (Figure 1).

In phase 2, patients had decreased normal spinal sagittal curvature due to decreased bone mineral density (BMD) and muscle mass. In phase 3, patients had advanced spinal sagittal malalignment due to aging and increased apparent muscle mass. In phase 4, patients had advanced irreversible spinal sagittal malalignment due to additional decreases in BMD and decreases in muscle mass. Because we included patients only in phases 2 and 3 in the current study, we observed that patients with a sagittal spinal malalignment tended to have greater muscle mass. Although further extensive study is needed, based on these findings, some intervention for skeletal muscle mass and spinal alignment in elderly osteoporosis patients who were independent and maintained their ADL might be recommended before decreasing in muscle mass and requiring surgeries for spinal deformity.

In the current study, BMI was higher in spinal malalignment group compared with normal group. Ando et al. reported obesity women showed spinal sagittal malalignment and worse health-related quality of life [18]. In addition, Eguchi et al. reported BMI was higher in patients with vertebral fractures compared with normal subjects [17]. Based on these findings obesity might be related with spinal sagittal malalignment and might lead to LBP in elderly osteoporosis patients. 

There were some limitations to the current study. First, as we mentioned above, an extensive inter-generational study and a larger sample size are needed. This would test our hypothesis about the four phases of aging and spinal alignment. Second, although we evaluated muscle mass in the current study, we did not measure muscle strength, including grip strength. There is a possibility that patients with spinal malalignment had lower muscle strength in spite of greater muscle mass.

## Conclusions

Elderly patients with osteoporosis and a sagittal spinal malalignment had more vertebral fractures and a higher risk of low back pain than patients with normal spinal alignment. Patients with a sagittal spinal malalignment who were independent and maintained their ADL showed high BMI and maintained muscle mass, independent of BMD and the number of vertebral fractures, contrary to our hypothesis. 
